# The Role of Cytoreductive Surgery with Hyperthermic Intraperitoneal Chemotherapy (HIPEC) in Peritoneal GIST-Induced Sarcomatosis (GISTosis)

**DOI:** 10.3390/jcm15020742

**Published:** 2026-01-16

**Authors:** John Spiliotis, Nikolaos Kopanakis, Athanasios Rogdakis, George Peppas, Aphrodite Fotiadou, Kyriacos Evangelou, Nikolaos Vassos

**Affiliations:** 1 Department of Peritoneal Surface Oncology, European Interbalkan Medical Centre, 57001 Thessaloniki, Greece; jspil@hotmail.gr (J.S.); peppasgr@yahoo.gr (G.P.); 2 Department of Surgery, Metaxa Cancer Memorial Hospital, 18537 Piraeus, Greece; n.kopanakis@metaxa-hospital.gov.gr; 3 Department of Surgery, St. Panteleimon General Hospital, Nikaia, 18454 Piraeus, Greece; rogdakis@gmail.com; 4 Department of General, Visceral and Transplantation Surgery, Heidelberg University Hospital, Ruprecht-Karls University of Heidelberg, 69117 Heidelberg, Germany; afrofotiadou386@gmail.com; 5 Department of Surgical Oncology, Athens Medical Center, 15125 Athens, Greece; evangeloukyriacos@gmail.com; 6 School of Medicine, European University Cyprus, Nicosia 2404, Cyprus; 7 Faculty of Medicine, Mannheim University Medical Center, University of Heidelberg, 68167 Mannheim, Germany

**Keywords:** hyperthermic intraperitoneal chemotherapy (HIPEC), GIST, peritoneal metastasis, peritoneal GISTosis, GIST-induced sarcomatosis

## Abstract

**Background:** The introduction of tyrosine kinase inhibitors has revolutionised the treatment of gastrointestinal stromal tumours (GISTs), yet the role of cytoreductive surgery (CRS) plus hyperthermic intraperitoneal chemotherapy (HIPEC) in peritoneal GISTosis remains controversial. **Methods:** A retrospective analysis was conducted on patients with peritoneal GISTosis who underwent CRS plus HIPEC in an 18-year period. We analysed the clinicopathological characteristics and evaluated the perioperative and long-term outcomes based on the extent of disease (peritoneal cancer index, PCI), the resection (completeness of cytoreduction score) and the IM-administration. The survival factors were also analysed and the Kaplan–Meier estimator to model and estimate overall (OS) and progression-free survival (PFS). The median follow-up period was 72 months (range, 12–146). **Results:** A total of 25 patients (M:F = 15:10) with a median age of 57 years (range, 32–69) underwent CRS with HIPEC for peritoneal GIST metastases, detected either synchronously (n = 11) or metachronously (n = 14). The media PCI score was 9 (range, 4–20) and complete cytoreduction was achieved in 80%. Grade III complications were observed in two patients, whereas there was no postoperative mortality. Neoadjuvant imatinib-mesylate (IM) therapy was administered in 60% of patients who detected with metachronous metastases (n = 8/14), whereas adjuvant IM therapy was administered in 19 of 25 patients. Median OS was 62 months (95% CI = 22.8–101.2). Median OS and DFS for patients with PCI scores ≤ 10 were significantly longer compared to those with PCI scores > 10 (*p* = 0.009 and *p* = 0.024, respectively). Patients with CC scores of 0–1 had a significantly longer OS compared to those with CC scores of 2 (*p* = 0.005) and 3 (*p* = 0.002) and longer PFS compared to those with CC scores of 3 (*p* = 0.005). The need for imatinib did not significantly impact OS (*p* = 0.240) or PFS (*p* = 0.243). **Conclusions:** CRS combined with HIPEC shows promising results in peritoneal GISTosis, especially in patients with lower PCI and CC scores. Until larger studies validate its safety and efficacy, it should be primarily performed in expert hands in specialised peritoneal surface oncology centres.

## 1. Introduction

Gastrointestinal stromal tumours (GIST) are rare tumours, yet the most common mesenchymal tumours of the digestive tract. Their clinical presentation depends on the tumour size and localisation and varies from small incidentalomas to large abdominal masses, which may present with a great variety of symptoms, such abdominal pain and gastrointestinal bleeding. Liver and peritoneum are the most common sites of GIST distant metastases, followed by lymph nodes, lungs or extraintestinal regions even more rarely [1,2,3]. Peritoneal metastasis is generally found as multiple peritoneal dissemination [4].

Hirota et al., with their discovery of a gain-of-function mutation in the KIT oncogene in 1998, shed light onto GISTs to better understand them and therefore develop treatment strategies [5]. The majority of GISTs (75%) present mutations in the c-KIT proto-oncogene and 10% in the platelet-derived growth factor receptor alpha (PDGFR-α). The remaining “wild-type” GISTs have a variety of other mutations and epimutations that may affect the SDH pathway [2,6]. Surgical resection remains the mainstay of treatment for localised GISTs. However, the introduction of tyrosine kinase inhibitors (TKIs) in 2002, which have become the paradigm of molecular therapy against cancer, has altered the management of localised and metastatic GISTs and offered prolonged survival to the patients with GISTs [4,7].

In the management of recurrent, locally advanced or metastatic GISTs, the use of imatinib is recommended alongside more aggressive cytoreductive surgery (CRS). As for so-called “peritoneal GISTosis” (PS) by Sugarbaker, there is currently no such indication of vast peritonectomy procedures for radical debulking [8]. Thus, the role of surgery for metastatic GISTs in the post-TKI era remains controversial. Recently, investigators have described the positive impact of resection in selected patients with metastatic GISTs, with mostly isolated peritoneal or liver metastases [9]. Taking also into account that GIST-induced sarcomatosis is chemotherapy-resistant, an appropriate treatment strategy for peritoneal GISTosis has not yet been established.

Despite the introduction of combined CRS with hyperthermic intraperitoneal chemotherapy (HIPEC) in the early 1980s, there are no universally accepted guidelines specifically for patients with peritoneal GISTosis. However, recent multicentre studies demonstrate promising survival results, therefore reassuring the clinicians about the technique’s safety and efficacy [10]. Particularly in the case of resistance to TKIs’ peritoneal metastases of GISTs, CRS combined with HIPEC might be considered, though evidence is limited primarily to case reports and small cohorts [11,12]. Especially in cases of peritoneal metastases from rare tumours, the clinical indications of combined CRS with HIPEC are usually based on the extrapolation of data from other malignancies treated with CRS and HIPEC and the individualised clinical experience [13].

Here we present a retrospective analysis of our approach, aiming to determine the surgical outcomes of CRS-HIPEC procedures on patients with GIST-induced intraperitoneal sarcomatosis who have been treated with TKIs, and to define the impact of TKI treatment on the overall survival of patients treated with CRS-HIPEC. A systematic review of the literature is also provided.

## 2. Materials and Methods

### 2.1. Patient Selection and Data Collection

A retrospective study was performed, recruiting data from GIST patients with peritoneal metastases who were treated from 2005 to 2023 in a large tertiary centre. All patients diagnosed with peritoneal GISTosis of metastatic origin and treated with CRS plus HIPEC were included in our cohort. Patients were selected for CRS+HIPEC based on multidisciplinary tumour board evaluation. A complete case analysis was used and patients with incomplete perioperative or survival data were excluded. The clinicopathological data of these patients were collected and summarised in a retrospective analysis. Data such as age (years), sex (male or female), primary tumour location (stomach, small intestine, or large intestine), symptom onset from initial treatment (earlier than 12 months, between 12 and 24 months, or later than 24 months), imatinib requirement (yes or no), main symptoms of peritoneal dissemination (intestinal obstruction, abdominal pain, gastrointestinal bleeding and/or fever), peritoneal cancer index (PCI) score and completeness of cytoreduction (CC), postoperative morbidity and mortality and follow-up data as well were retrieved.

### 2.2. Operative and Therapeutic Characteristics

The treatment plan of each patient was managed by a multidisciplinary GIST team. Before surgical or TKI treatment started, tumour biopsy was obtained and diagnosis of the GIST was confirmed. The need for TKI treatment was documented for all patients. The type of TKI treatment was based on the mutational analysis and the TKI treatment duration was intended to last at least 6 months or as long as the tumour was still shrinking in size. Response to IM therapy was evaluated 1 month after the treatment start and then every 3 months either with positron emission computed tomography (PET-CT), dual-energy computed tomography (DE-CT) or contrast-enhanced magnetic resonance (CE-MRI). Response was determined according to the Response Evaluation Criteria In Solid Tumors version 1.0 and version 1.1 (RECIST 1.1) as a complete response (CR), partial response (PR), stable disease (SD) or progressive disease (PD) [14].

HIPEC was performed after CRS in accordance with the technique described by Sugarbaker et al., aiming to resect all macroscopic visible peritoneal disease and to attain a complete cytoreduction [15]. Chemoperfusion was performed using the closed technique for 60 min at 40 C and cisplatin (50 mg/m^2^) as the chemotherapeutic agent. To quantify the extent of peritoneal GIST spread, the peritoneal cancer index (PCI) score was calculated and utilised as a prognostication tool. The completeness of cytoreduction (CC) score was also employed for the assessment of residual disease postoperatively.

### 2.3. Statistical Analysis

Statistical analysis performed using the SPSS v23.0 software. Due to the retrospective nature of the study, confounder control was limited to stratified analyses; no propensity score matching was possible. Survival outcomes were analysed. Overall survival (OS) and disease-free survival (DFS) were the endpoints of interest for survival analysis. OS was defined as the interval between the date of surgery and the date of death from any cause, while DFS was defined as the interval between the date of surgery and the date of 1. local recurrence evidence, 2. regional recurrence evidence, 3. distant metastasis evidence or 4. death from any cause (coinciding with OS). OS and DFS curves were estimated using the Kaplan–Meier method [16,17]. All *p*-values are two-tailed and statistical significance was set at *p* = 0.05.

### 2.4. Systematic Review

A systematic-wise approach was followed to identify previous studies that submitted patients with peritoneal GISTosis to HIPEC with or without CRS.

Studies reporting at least one case of peritoneal GISTosis with at least one of the following variables were included: gender, location of primary tumour, interval between the initial treatment finalisation and the onset of symptoms, use of imatinib, symptoms of peritoneal disease, chemotherapeutics utilised for HIPEC, overall survival, disease-free survival, morbidity and mortality. Publications whose full texts were unavailable or in any language besides English (with an English translation not provided) were excluded. There were three final inclusion groups:

1. The first group included all studies that reported cases of peritoneal sarcomatosis, regardless of histological subtype; sarcomas were considered as such according to the third volume of the fifth edition of the World Health Organisation (WHO) series on the classification of human tumours 2020 (Soft Tissue and Bone Tumours) [18].
2. The second group included those studies from the first group that reported cases of peritoneal GISTosis in particular, regardless of the use of HIPEC with or without CRS.
3. From all studies comprising the second group, those that reported the use of HIPEC with CRS for the treatment of peritoneal GISTosis were included in the third group.

PubMed, Embase, Scopus and Cochrane Library were consulted for potentially eligible study identification. The following search strings were used according to database:

- PubMed: “Peritoneal Sarcomatosis” OR (“Peritoneal Neoplasms”[MeSH] AND (“Gastrointestinal Stromal Tumors”[MeSH] OR “GIST” OR “Gastrointestinal Stromal Tumour*”)).
- Embase: ‘Peritoneal Sarcomatosis’ OR (‘Peritoneal Neoplasms’/exp AND (‘Gastrointestinal Stromal Tumors’/exp OR ‘GIST’ OR ‘Gastrointestinal Stromal Tumour’)).
- Scopus: “Peritoneal Sarcomatosis” OR (“Peritoneal Disease” AND (“GIST” OR “Gastrointestinal Stromal Tumors” OR “Gastrointestinal Stromal Tumours” OR “Gastrointestinal Stromal Tumor” OR “Gastrointestinal Stromal Tumour”)).
- Cochrane Library: “Peritoneal Sarcomatosis” OR (“Peritoneal Neoplasms”[MeSH] AND (“Gastrointestinal Stromal Tumors”[MeSH] OR “GIST” OR “Gastrointestinal Stromal Tumour*”)).

A supplemental approach was followed to manually identify additional primary studies that were potentially eligible for inclusion through hand-searching and reference list inspection.

Rayyan was used as a web-tool to facilitate title and abstract screening and sharing and tracking of decisions. Initial screening was processed by two independent reviewers and conflicts were resolved following consensus-based discussion and the participation of a third party.

Gender, location of the primary tumour, interval between the initial treatment finalisation and the onset of symptoms, use of imatinib, symptoms of peritoneal disease, chemotherapeutics utilised for HIPEC, overall survival, disease-free survival, morbidity and mortality were noted in summary for each article and included in a contingency table. In cases where data for peritoneal GISTosis were not provided individually, relevant information for all patients with carcinomatosis, sarcomatosis, or peritoneal disease attributed to a subgroup of sarcomas was alternatively reported and indicated as such. Missing data or data not explicitly documented were referred to as not specified.

**Figure 1:**
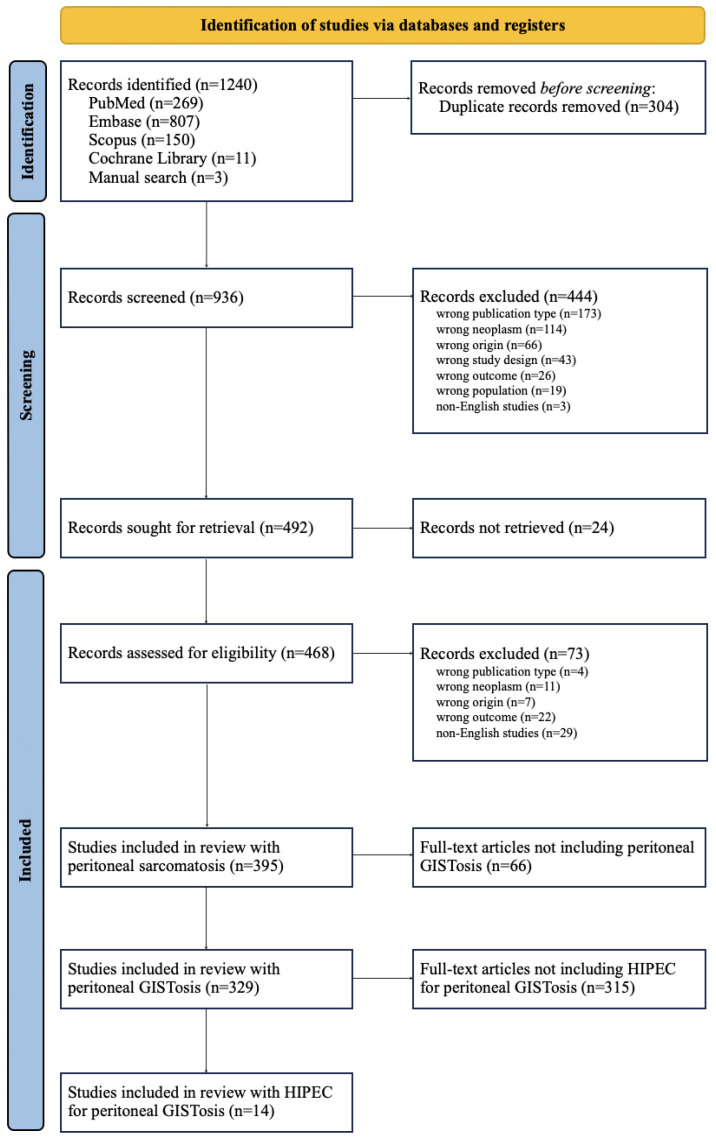
PRISMA flowchart summarising the results of the search and selection process.

The study selection process is summarised in Figure 1. From a total of 1240 records, 395 studies were eligible and 14 were included in the data analysis [19,20,21,22,23,24,25,26,27,28,29,30,31,32]. No formal bias assessment (e.g., ROBINS-I or NOS) was conducted.

## 3. Results

### 3.1. Original Study

#### 3.1.1. Clinical Characteristics

Thirty-five CRS plus HIPEC procedures in 25 patients with peritoneal GISTosis were performed between 2005 and 2023 at our institution. The majority of patients were male (n = 15; 60.0%) and the mean age was 57 years (range: 32–69 years). Most primary tumours originated from the stomach (n = 10; 40.0%), followed by the small intestine (n = 8; 32%). The peritoneal metastases were detected synchronously with the primary tumour in 11 cases (44%), whereas in 14 cases (56%), the metastases were detected metachronously in a median period of 18 months (range, 12–36), following resection of the primary tumour without rupture. The most common symptoms of the peritoneal GIST dissemination were intestinal obstruction, abdominal pain and gastrointestinal bleeding. The demographic and clinical characteristics are summarised in Table 1.

#### 3.1.2. Therapeutic Characteristics

Regarding the CRS and HIPEC procedure, the median PCI score during the CRS/HIPEC was 9 (range, 4–20), and complete cytoreduction (CC-0/1) was achieved for the majority of the patients (n = 20; 80%); the five patients were able to achieve a CC-2 score. The length of surgery was 427 min (range, 239–617) and the required ICU time was 1.2 days whereas the median hospital stay on the surgical floor was 12 days (range, 7–17).

Postoperative complications were presented in eight patients, with most of them (n = 6; 24%) undergoing grade I complication. Importantly, grade III complication was observed only in two patients (8%). More specifically, major complications encompassed enterocutaneous fistulae, which resolved spontaneously following total parenteral nutrition (TPN) on the 22nd and 40th postoperative day, respectively. Further details about the postoperative morbidity are presented in Table 2. No postoperative mortality was observed.

Almost half of the patients who underwent CRS (incl. resection of primary tumour) and HIPEC for synchronous peritoneal metastases received adjuvant tyrosine kinase inhibitor (TKI) treatment. On the other hand, when peritoneal metastases were detected metachronously, a TKI treatment for a median period of 6 months was administered in eight patients (n = 8/14, 60%), whereas the other patients were directly led to CRS and HIPEC because of the low preoperative PCI. However, all patients with metachronous metastases were treated with adjuvant TKI treatment after the CRS/HIPEC procedure.

#### 3.1.3. Survival

Median follow-up was 72 months (range, 12–146). Four patients were lost to follow-up. During follow-up, among the 21 patients included in the calculations, 13 patients (76%) were alive without evidence of disease and under TKI treatment, 5 patients were alive with stable disease under TKI treatment, 2 patients died of disease despite TKI treatment, whereas 1 patient died due to other reasons.

The median unstratified overall survival was established at 62 months, with a 95% CI of 22.8–101.2 months.

**Figure 2:**
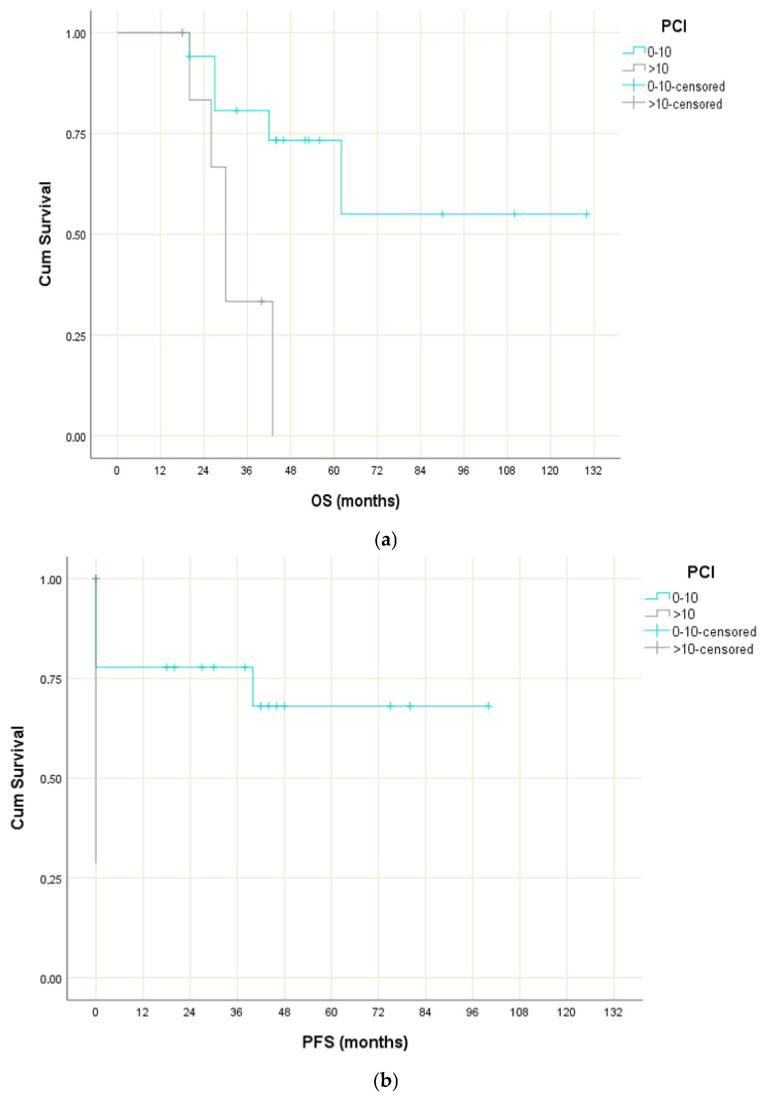
(**a**). Overall survival function of patients with peritoneal GISTosis undergoing CRS plus HIPEC according to their peritoneal cancer index score (PCI ≤ 10 vs. PCI > 10). (**b**). Progression-free survival of patients with peritoneal GISTosis undergoing CRS plus HIPEC according to their peritoneal cancer index score (PCI ≤ 10 vs. PCI > 10).

Τhere was no significant difference in OS between men and women (*p* = 0.934), between patients aged <50 and ≥50 years (*p* = 0.698), and between patients with primary gastric and small intestinal tumours (*p* = 0.322) or gastric and colon tumours (*p* = 0.523). Interestingly, the OS for patients with PCI scores ≤ 10 was significantly longer compared to patients with PCI scores > 10 (χ^2^(1) = 6.866; *p* = 0.009) (Figure 2a), and furthermore, the median OS for patients with CC scores equal to 0 or 1 was significantly longer compared to patients with CC scores of 2 (χ^2^(1) = 7.776; *p* = 0.005).

Similarly, no significant difference in progression-free survival (PFS) when gender (*p* = 0.985), age (*p* = 0.574), tumour site (*p* = 0.464), CC score (*p* = 0.068) and TKI-treatment (*p* = 0.243) were analysed, but not concerning the PCI and CC scores. More specifically, the median PFS for patients with PCI scores ≤ 10 was significantly longer compared to patients with PCI scores > 10 (χ^2^(1) = 5.085; *p* = 0.024) (Figure 2b). Similarly, the median PFS for patients with CC scores equal to 0 and 1 was significantly longer compared to patients with CC scores of 3 (χ^2^(1) = 7.824; *p* = 0.005).

### 3.2. Previous Studies

#### 3.2.1. General Study Characteristics

The total 14 studies were conducted in the USA (5), Italy (4), Spain (3), France (1), Saudi Arabia (1) and Turkey (1). All studies were published after 2004. Study characteristics are summarised in Table 3. Among the 14 studies, 13 were classified as prospective studies (PSs) and one was a report of a case. All studies assessed the efficacy of radical CRS-HIPEC in treatment of peritoneal sarcomatosis (mostly visceral or non-visceral GISTs and gynaecological sarcomas) in different time frames, ranging from 1 to 26 years.

#### 3.2.2. Study Outcomes

The reported outcomes from the studies included in this review were analysed and thus are presented in Table 3. (Median) overall survival (OS), (median) disease-free survival (RFS) and death-of-disease, morbidity and mortality rates were estimated and documented.

## 4. Discussion

Recent evidence highlights the critical role of imatinib in the management of advanced and high-risk gastrointestinal stromal tumours (GISTs) [33]. In particular, high- compared to low-dose imatinib (400 mg twice daily vs. 400 mg once daily) in patients with high-risk GISTs is associated with improved progression-free survival (PFS), especially in patients with KIT exon 9 mutations, while the largest and most recent (2024) meta-analysis on neoadjuvant imatinib administered in locally advanced and metastatic GISTs revealed that extended (3 years) adjuvant low-dose therapy demonstrates superior recurrence-free survival (RFS) and overall survival (OS) compared to 1-year administration [34,35]. A randomised trial comparing two doses of imatinib in almost 1000 patients with advanced GISTs determined initial resistant rates to 12.4%; lung metastases, the absence of liver metastasis, increased granulocyte count and decreased haemoglobin levels were identified as independent initial resistance predictors, while high baseline granulocyte count, extra-gastric location of primary tumour, large size and low initial imatinib dose were recognised as independent predictors of late imatinib resistance [36].

Locoregional surgical treatment of peritoneal GISTosis, defined as cytoreductive surgery (CRS) or ablation, is reported to generate 2- and 5-year OS rates as high as 90% (95% CI: 83–98%) and 63% (95% CI: 48–80%), respectively, according to a 2023 multicentre analysis from the Dutch GIST registry [37]. Conjointly with adjuvant imatinib, CRS can prolong DFS [38]; this was confirmed by Bryan et al., who determined a median survival of 7.89 years vs. 1.04 years in patients who underwent CRS+HIPEC with or without (neo)-adjuvant TKIs, respectively [25]. Notably, Xinhua et al. compared patients who received continuous circulatory HIPEC (C-HIPEC), using mitoxantrone or doxorubicin plus cisplatin, versus non-C-HIPEC, following CRS for peritoneal GISTosis. Median OS and PFS were 40 and 16 months, respectively, with no statistically significant differences in median OS (35 months vs. 37 months; *p* = 0.6187) or PFS (13 months in both groups; *p* = 0.9865). Postoperative grade III-IV 30-day morbidity was 16.3% overall, with no significant between-group difference (14.3% vs. 17.4%; *p* = 0.901). In this setting, the use of cisplatin-based HIPEC in our study represents an adjunctive strategy aiming to reduce the risk of peritoneal recurrence by targeting microscopic residual disease, in analogy to protocols applied in other peritoneal surface malignancies, rather than a direct cytotoxic efficacy against GIST cells.

A four-centre study from the European Organisation for Research and Treatment of Cancer Soft Tissue and Bone Sarcoma Group (EORTC-STBSG) investigated the outcomes of metastasectomy in patients with metastatic GISTs, 64.4% of whom had also developed peritoneal GISTosis [39]. Multivariate analysis revealed resection status (R2 vs. R0/R1) as an independent prognostic factor for OS (HR = 2.26; 95% CI = 1.09–4.68; *p* = 0.001), although the number of resected metastases (2–4 vs. 1 and ≥5 vs. 1) was interestingly not prognostic (HR = 0.68; 95% CI = 0.25–1.68 and HR = 1.05; 95% CI = 0.44–2.49, respectively; *p* = 0.506). Along these lines, our analysis has corroborated that CC 0–1 scores are associated with significantly improved OS compared to both CC = 2 (χ^2^(1) = 7.776; *p* = 0.005) and CC = 3 (χ^2^(1) = 9.427; *p* = 0.002) scores, and better DFS compared to CC = 3 scores (χ^2^(1) = 7.824; *p* = 0.005). Thus the importance of state-of-the-art surgery in specialised centres by experienced surgeons is demonstrated, as it reduces the burden of the disease, especially when it comes to localised peritoneal metastases.

The combination of CRS with adjuvant imatinib for at least 3 years is the standard treatment for high metastatic risk GISTs, as it improves recurrence rates and OS as evidenced by the SSGXVIII/AIO open-label, multicentre, randomised, phase III trial [38]. Overall, as imatinib treatment seems to be a stronger predictor of survival compared to CRS, the latter should be reserved as an adjuvant option for those who demonstrate either favourable tumour biology (as indicated by imatinib treatment response) or minimal disease progression post-response [36].

Neoadjuvant imatinib treatment is particularly valuable in patients with metachronous metastases, except for cases with low cPCI, where primary resection of the metachronous metastasis plus adjuvant imatinib is considered effective treatment [40]. Multiple studies have demonstrated that R0/R1 margins following CRS are more beneficial for patients who respond to imatinib compared to those with progressive disease [36,41,42,43,44,45]. Synchronous peritoneal metastases, in our study and in most previous studies, especially by emergency resections or by very few lesions, were often incidental findings during primary tumour resection [46]. Consequently, these patients have not received neoadjuvant imatinib as part of their initial treatment plan [47].

Our analysis distinguishes itself from previous studies by presenting the largest cohort of patients with peritoneal GISTosis who underwent CRS+HIPEC to date. Our literature review identified nearly 400 studies on peritoneal sarcomatosis, but only 16.7% focused on peritoneal GISTosis. Among the latter, just 4.3% included HIPEC as part of the treatment strategy, resulting in only 3.5% of peritoneal sarcomatosis manuscripts being related to HIPEC for peritoneal GISTosis.

It is important to recognise that the primary driver of the improved outcomes in this study is likely the completeness of cytoreduction, as indicated by the strong correlation between CC scores and survival. Due to the retrospective nature of the study and the lack of a TKI-only comparator group, we cannot isolate the individual contribution of HIPEC. Future studies are needed to compare outcomes between patients treated with TKI alone versus those undergoing cytoreductive surgery, ideally in multicentric or prospective registry settings.

As a result, knowledge about HIPEC-associated benefits in patients with peritoneal GISTosis is limited. Therefore peritonectomy with multiple visceral resections, whenever indicated, should be preferred while HIPEC reserved for low PCI cases [48] and in the hands of experts in specialised peritoneal surface oncology centres [49], with debulking or palliative surgery limited to patients with peritoneal GISTosis-induced complications (e.g., haemorrhage, abdominal pain, ileus) [12].

## 5. Conclusions

Cytoreductive surgery (CRS) with hyperthermic intraperitoneal chemotherapy (HIPEC) can be a valuable tool for the management of peritoneal GISTosis. Our study demonstrated an acceptable safety profile and favourable outcomes in terms of overall survival (OS) and disease-free survival (DFS) in the largest cohort of CRS+HIPEC for peritoneal GISTosis to date, particularly in patients with low peritoneal cancer index (PCI) and completeness of cytoreduction (CC) scores. The need for imatinib therapy did not significantly impact survival outcomes. With careful patient selection and optimal surgical techniques in specialised peritoneal surface oncology centres, CRS-HIPEC offers the potential for improved outcomes and enhanced quality of life compared to conventional peritonectomy or TKI therapy alone. Specifically, the utility of HIPEC itself in those settings remains uncertain and should be explored further in prospective, controlled settings. Until then, it should be regarded as an adjunct to complete cytoreduction in expert centres, rather than a proven oncologic necessity. Further research through larger studies is necessary to validate these findings and better delineate the safety profile and feasibility of CRS-HIPEC in peritoneal GISTosis.

## Figures and Tables

**Table 1:** Overall demographic and clinical characteristics of all 25 patients who underwent CRS plus HIPEC for peritoneal GISTosis.

Characteristic		No. (%)
Sex		
	Male	15 (60.0)
	Female	10 (40.0)
Location		
	Stomach	10 (40.0)
	Small intestine	8 (32.0)
	Large intestine	7 (28.0)
Imatinib requirement		
	Yes	15 (60.0)
	No	10 (40.0)
Main symptoms of peritoneal dissemination		
	Intestinal obstruction	9 (36.0)
	Abdominal pain	7 (28.0)
	Gastrointestinal bleeding	6 (24.0)
	Fever	3 (12.0)

**Table 2:** Overall morbidity and mortality rates for the total of patients (n = 25).

Measure of Disease Burden			No. (%)
Morbidity			
	Major complications		
		Enterocutaneous fistulae	2 (8.0)
	Minor complications		
		Catheter-related sepsis	3 (12.0)
		Pleural effusions	2 (8.0)
		Urine tract infections (UTIs)	1 (4.0)
Mortality			
	Postoperative deaths		0

**Table 3:** Summary of studies reporting the use of HIPEC for peritoneal GISTosis and their demographic, clinical and outcome-related characteristics.

Author (Date) Total Patient Number (tN) GIST N (%)	Demographic, Clinical and Outcome-Related Variables									
	Gender	Location	Symptom Onset from Treatment	Need for Imatinib	Main Symptoms of Peritoneal Disease	HIPEC Drugs	OS	DFS	Morbidity	Mortality
Spiliotis et al. (Current Study) tN = 25 25 (100.0)	Male: 15 Female: 10	Stomach: 10 Small intestine: 8 Large intestine: 7	Earlier than 12 months: 6 12–24 months: Later than 24 months: 9	Treated with imatinib: 15 no imatinib treatment: 10	Intestinal obstruction: 9 Abdominal pain: 7 Gastrointestinal bleeding: 6 Fever: 3	Cisplatin	Median OS: 62 months	Median DFS: 13 months	CD III: 2	0
Casares et al. (2024) [20] tN = 23 6 (26.1)	Male: 7 * Female: 16 *	Viscera: 6	NS	NS	NS	Doxorubicin: 4 * Doxorubicin + cisplatin: 16 * Paclitaxel: 3 *	3-year OS: 100.0%	33.3% (3-year)	CD III-IV: 3 *	0
Duzgun et al. (2019) [22] tN = 20 1 (5.0)	Male: 13 * Female: 7 *	NS	NS	The tumour is reported to be resistant to imatinib	NS	Doxorubicin + cisplatin	NS	Recurrence (unspecified)	CD I: 6 * CD II: 9 * CD IIIa: 2 * CD IIIb: 2 * CD V: 1 *	0
Goéré et al. (2017) [23] tN = 781, however only 734 included in survival analysis 47 (6.0)	Male: 516 * Female: 218 *	NS	NS	NS	NS	Cisplatin: 329 * Doxorubicin: 134 * Mitomycin-c: 319 * Irinotecan: 33 * Oxaliplatin: 130 * Doxorubicin + cisplatin: 113 * Mitomycin-c + cisplatin: 82 *	HR = 1.43 (0.72–2.84), *p* < 0.0001 Median OS: 39.5 months * 1-year OS: 77.8% * 3-year OS: 52.2% * 5-year OS: 38.7% *	Median 5-year DFS: 14.3% † 1-year DFS: 61.2% * 3-year DFS: 33.2% * 5-year DFS: 28.0% *	NCI CTCAE V4.0 III-IV: 272 *	19 *
Abu-Zaid et al. (2016) [24] tN = 11 2 (18.2)	Male: 9 * Female: 2 *	Fundus of the stomach: 1 Body of the stomach: 1	NS	NS	Asymptomatic: 1 * Flank pain: 2 * Abdominal pain: 8 * Increased abdominal circumference: 2 * Early satiety, nausea and vomiting: 2 * Weight loss: 3 *	Doxorubicin + cisplatin: 6 * Melphalan: 4 * Mitomycin-c: 1 *	Median OS: 28.3 ± 3.2 months *	Median: 18.0 ± 4.0 months *	CD I: 1 * CD II: 3 * CD IVa: 1 *	2 *
Cardi et al. (2015) [25] tN = 28 3 (10.7)	Male: 1 Female: 2	NS	NS	All tumours reported to be resistant to imatinib	NS	Cisplatin: 3	Median OS: 56 months * 5-year OS: 40.3% *	ADF at 34 months: 1 ADF at 108 months: 1	CD I/II: 6 * CD III: 2 * CD IV: 4 *	1
Bryan et al. (2014) [26] tN = 16 16 (100.0)	Male: 8 Female: 10	Stomach: 5 Small intestine: 10 EGIST: 1	NS	NS	NS	Mitomycin-c ± mitoxantrone	Median OS: 3.33 years 3-year OS: 56.0%	NS	CD I/II: 8 CD III/IV: 6 CV V: 1	1
Medina Fernández et al. (2014) [2] tN = 1 1 (100.0)	Female	Jejunum	3 months	Resistant to imatinib	NS	Doxorubicin + cisplatin	Alive at 6 months post- surgery	No evidence of recurrence at 6 months post-surgery	None	0
Sommariva et al. (2013) [27] tN = 15 2 (13.3)	Male: 1 Female: 1	Stomach: 2	NS	No patient was treated with imatinib	NS	Mitomycin-c + cisplatin: 2 * Doxorubicin + cisplatin: 13 *	Median OS: 27.0 months * 1-year OS: 80.0% * 3-year OS: 36.0% * 5-year OS: 29.0% *	ADF at 134 months: 1	CD III-IV: 7	1
Baumgartner et al. (2013) [28] tN = 15 2 (13.3)	Male: 8 * Female: 7 *	RP: 4 * Peritoneal: 4 * Pelvis: 5 * Extra- abdominal: 2 *	NS	Both patients treated with imatinib	Asymptomatic: 11 *	Mitomycin-c: 14 * Doxorubicin: 1 * Cisplatin: 2 *	Median: 23.9 months	Median: 17.2 months *	CD III-IV: 4 *	0
Konstantinidis et al. (2012) [29] tN = 14 1 (7.1)	Male: 7 * Female: 7 *	NS	NS	NS	NS	Mitomycin-c	DOD after 1 month: 1 ‡ DOD after 6 months: 1 ‡	AWD 5 months post- operatively: 1 ‡	CD III: 4 *	2 †
Baratti et al. (2010) [21] tN = 37 8 (21.6)	Male: 4 Female: 4	NS	NS	After 2000, patients treated with imatinib	NS	Cisplatin + mitomycin: * Cisplatin + doxorubicin: *	Median OS: 20 months for GIST metastasis (before 2000) Median OS: 5 years for GIST-meta (after 2000)	<6 months	21.6	3.7
Levine et al. (2014) [32] tN = 1000 9 (0.9)	Male: 469 * Female: 531 *	Peritoneal surface: 1000	NS	Treated with imatinib	NS	Mitomycin c +/− mitoxantrone: 9	Median OS: 29.4 months *	5-year DFS: 32.5% †	30-day morbidity rate = 42% *	30-day morbidity rate = 3.8% *
Lim et al. (2007) [30] tN = 28 17 (58.6) §	Male: 15 * Female: 13 *	NS	NS	NS	Asymptomatic: 11 * Abdominal pain: 14 * increased abdominal girth: 7 * Nausea, emesis, early satiety: 3 * Weight loss: 2 * Fatigue: 1 *	Cisplatin: 15 § Mitoxantrone + cisplatin: 2 §	Median OS: 16.9 months (cisplatin)/5.5 months (mitoxantrone + cisplatin) *	Median DFS: 4.4 months (cisplatin)/2.3 months (mitoxantrone + cisplatin) * AWD at 41 months: 1 AWD at 67 months: 1 * AWD at 68 months: 1 * ADF at 68 months: 1 *	30-day surgical complications: 7 * NCI toxicity III/IV: 83 *	24 *
Rossi et al. (2004) [31] tN = 60 14 (23.3)	Male: 25 * Female: 35 *	NS	NS	NS	NS	Doxorubicin + cisplatin	Median OS: 36.0 months *	Median DFS: 24.0 months * AWD: 12 * ADF: 16 *	Morbidity rate = 33.0% *	32 *

* Data for both GIST- and non-GIST-induced peritoneal dissemination cases. † Data reported for the whole sarcoma group. ‡ Data reported for three patients who underwent palliative cytoreductive surgery with hyperthermic intraperitoneal chemotherapy. § GIST and leiomyosarcoma patients reported jointly as most were diagnosed prior to routine c-kit testing; ADF = alive disease-free; AWD = alive with disease; CD = Clavien–Dindo class; DFS = disease-free survival; DOD = died of disease; EGIST = extra-gastrointestinal stromal tumour; GIST = Gastrointestinal Stromal Tumour; HIPEC = hyperthermic intraperitoneal chemotherapy; HR = hazard ratio; NCI-CTCAE = National Cancer Institute Common Terminology Criteria for Adverse Events Version 4.0; NS = information not specified; OS = overall survival; RP = retroperitoneum.

## Data Availability

The data presented in this study are available upon reasonable request from the corresponding author.
